# Identification of three novel *TCOF1* mutations in patients with Treacher Collins Syndrome

**DOI:** 10.1038/s41439-021-00168-4

**Published:** 2021-09-27

**Authors:** Bożena Anna Marszałek-Kruk, Piotr Wójcicki

**Affiliations:** 1 Department of Genetics, Wrocław University of Environmental and Life Sciences, Wrocław, Poland; 2 Wrocław Medical University, Wrocław, Poland

**Keywords:** Molecular biology, Medical genetics, Mutation, Diseases, Disease genetics

## Abstract

Here we describe three novel *TCOF1* mutations found in unrelated patients with Treacher Collins syndrome. These mutations include one deletion, NM_001135243.2:c.2604_2605delAG (p.Gly869Glufs*3), and two substitutions, NM_001135243.2:c.2575C>T (p.Gln859*) and NM_001135243.2:c.4111G>T (p.Glu1371*). These mutations cause shortening of a protein called Treacle in patients with features typical of TCS. Continuous identification of new mutations is important to expand the mutation base, which is helpful in the diagnosis of both patients and their families

Treacher Collins syndrome (TCS, OMIM 154500), also called mandibulofacial dysostosis (MFD), was described in 1900 and extensively examined by Franceschetti and Klein in 1949^1^. It is a rare developmental disorder with an incidence rate of ~1 in 50,000 live births. Forty percent of cases are related to family history, whereas the remaining incidences occur due to de novo mutations. The literature describes four clinical TCS subtypes: TCS1 (OMIM 154500) is a result of mutations in the *TCOF1* gene (OMIM 606847), TCS2 (OMIM 613717) is caused by mutations in the *POLR1D* gene (OMIM 613715), TCS3 (OMIM 248390) is caused by *POLR1C* gene mutations (OMIM 610060), and TCS4 (OMIM 618939) is caused by mutations in the *POLR1B* gene (OMIM 602000). *TCOF1* and *POLR1B* sequence variants have autosomal dominant inheritance, variants in *POLR1C* are autosomal recessive, and variants in *POLR1D* can be either autosomal dominant or autosomal recessive. The *TCOF1* gene has been cloned by the Treacher Collins Syndrome Collaborative Group^2^. It maps to 5q32-q33.1. So et al.^3^ discovered two additional exons: 6A (231 nt) and 16A (108 nt). Some of the reported transcripts lack exon 19 (114 nt). Dauwerse et al.^4^ detected mutations in the *POLR1C* gene, which maps to 6p21.1, whereas *POLR1D* maps to 13q12.2 in TCS patients. Sanchez et al.^5^ demonstrated that *POLR1B* mutations located on 2q14.1 were also found in patients with TCS. *TCOF1* gene mutations are responsible for ~86% of TCS cases, *POLR1C*: 1.2%, *POLR1D*: 6%, and *POLR1B*: 1.3%^4–6^. *TCOF1* encodes a 144 kDa nuclear phosphoprotein called Treacle, which is composed of 1488 amino acids^7^.

Mutations in *TCOF1* may lead to haploinsufficiency due to the formation of a shortened protein. Treacle contains three domains, of which the central domain is the largest, embodying ten repeats of serine clusters subdivided by stretches rich in the amino acids alanine, proline, lysine, and glutamic acid^8^. Ciccia et al.^9^ studied the interaction between the DNA damage response factor (DDR) and *TCOF1*. This gene plays a regulatory role as a DDR factor and Treacle forms part of the NBS1 protein complex. The N and C termini of Treacle contain nuclear export and import signals, which is evidence of its dynamic localization^10^. Protein localization is related to the LisH motif, which exists in the N-terminal domain^11^. Treacle plays an meaningful role in proper rDNA transcription during ribosome biogenesis^7^ and mitosis^12^.

More than 200 mutations have been reported in *TCOF1*^13–15^. The majority of these mutations are small deletions that result in a premature termination codon, which produces a truncated protein. Bowman et al.^16^ identified multiple pathogenic *TCOF1* gene rearrangements of different sizes at a rate of 5% in examined unrelated individuals with TCS. Beygo et al.^17^ used multiplex ligation‐dependent probe amplification to identify a large 3.367 kb deletion eliminating the entire exon 3 in one patient, suggesting that the rate of large deletions was low at <1%. Vincent et al.^6^ described large 1 Mb and 262 kb mutations resulting in complete gene deletions of *TCOF1* and *CAMK2A*. No correlation was observed between the type of mutation and patient phenotype. The management of patients requires a multidisciplinary treatment team involving pediatricians, otolaryngologists, clinical geneticists, orthodontists, audiologists, and surgeons.

In this study, we presented three novel mutations (two in exon 16 and one in exon 24) of the *TCOF1* gene in MFD patients.

The described examinations were conducted according to guidance from an ethics committee and in accordance with the Declaration of Helsinki. Blood from affected individuals was collected after obtaining informed consent from the patients or their legal guardians.

Genetic material from three different, unrelated patients of Caucasian ethnicity was examined. The patients had commonly observed clinical features characteristic of TCS, such as downward-slanting palpebral fissures, mandibular hypoplasia, delayed speech development, and atresia of the external ear canal. Typically, TCS patients do not demonstrate cognitive disability. The phenotypic features of the examined patients are summarized in Table 1.

**Table 1:** Clinical characteristics of examined patients.

Examined patient	1	2	3	
**Clinical features**				**Occurrence in population**^6^
Downward-slanting palpebral fissures	X	X	X	89–100%
Malar hypoplasia	–	X	X	81–97%
Conductive hearing loss	–	X	X	83–92%
Mandibular hypoplasia/micrognathia	X	X	X	78–91%
Atresia of external ear canal	–	X	–	68–71%
Microtia	–	–	X	10–77%
Coloboma of the lower lid	–	–	–	54–69%
Asymmetry	X	X	–	52%
Preauricular hair displacement	X	–	–	24–49%
Cleft palate	–	–	–	21–33%
Respiratory problems	–	–	–	12–18%
Microcephaly	–	–	–	3%

“X” the patient has this feature; “-” feature not identified in patient

In all three cases, DNA was isolated from the peripheral blood leukocytes of patients and parents. In addition, blood was collected from the siblings of one patient. PCR reaction of exons 1–27 with adjacent intron fragments was then performed. PCR products were sequenced after the sample purification phase. Using appropriate primers for each exon, samples were analyzed by capillary gel electrophoresis based on the ABI 310 DNA System. The nomenclature for sequence variants used in our report is in accordance with Human Genome Variation Society (HGVS) guidelines. The National Center for Biotechnology Information (NCBI) database was used for verification of the mutations (https://www.ncbi.nlm.nih.gov/variation/tools/1000genomes/). The sequencing results were compared with *Homo sapiens* chromosome 5, referring to the NCBI reference sequence NM_001135243.2.gb transcript variant 4 for *TCOF1*. The exon position of the mutation, kind on amino acid involved in the change and predicted effect on the protein, were indicated by https://www.mutalyzer.nl/ and confirmed by http://www.mutationtaster.org/. The results were submitted to dbSNP (https://www.ncbi.nlm.nih.gov/snp/).

Sequence analysis allowed us to identify three mutations, one frameshift and two nonsense mutations, located in *TCOF1* hotspots. In patient 1, we found a heterozygous 2 bp deletion in exon 16 (NM_001135243.2:c.2604_2605delAG). In this case, an in-frame deletion caused the premature termination of translation at amino acid 871 (p.Gly869Glufs*3). This resulted in the formation of a protein product lacking a nuclear localization signal. Direct sequencing of the DNA from the patient’s siblings and parents did not show similar sequences. Compared with a wild-type sequence, the mutation in the proband was considered de novo. Sequence analysis of material from the second patient revealed a novel transition (NM_001135243.2:c.2575C>T) at (p. Gln859*) in exon 16. The predicted effect of this substitution is shortening of the Treacle protein. In the case of individual three, the substitution NM_001135243.2:c.4111G>T was found in exon 24 and resulted in a stop codon at (p.Glu1371*). Neither substitution was found in the parents of the respective probands, indicating de novo mutations. All three recognized sequence variants are heterozygous. The examination results are presented as chromatograms comparing patient and control sequences in Fig. 1.

**Fig. 1:**
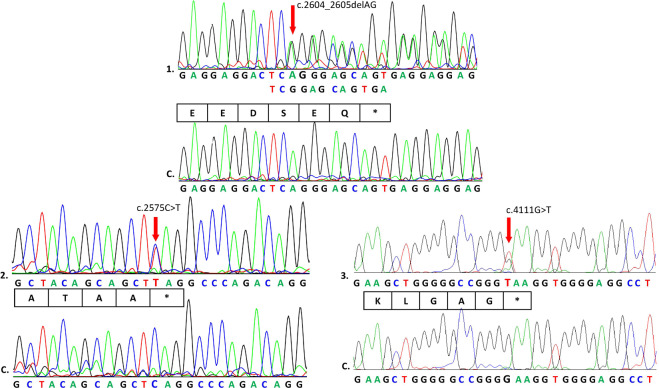
Analysis of *TCOF1* gene mutations. Sequence analysis of the amplified fragments of the *TCOF1* gene: 1, patient with c.2604_2605delAG in exon 16; 2, patient with c.2575C>T in exon 16; 3, patient with c.4111G>T in exon 24; and C, controls. Arrows indicate the location of mutations and stop codons are marked by *.

The majority of *TCOF1* mutations are deletions that range in size from 1 to 40 nucleotides, resulting in a truncated protein^13,18,19^. Most commonly recognized sequence variants are small insertions, splice site variants, and missense and nonsense variants (19–23% of mutations). All three variations detected here were in the coding sequence of the *TCOF1* gene. Exons 16 and 24 contain a portion of the coding sequence and encode the central domain of the Treacle protein. The central domain plays a significant role in protein structure, as it contains kinase C and casein kinase 2-phosphorylation sites. Therefore, the identified mutations may affect the abnormal conformation and dysfunction of the Treacle protein. Mutational hotspots in *TCOF1* include exons 10, 15, 16, 23, and 24. In this study, we present one deletion, NM_001135243.2:c.2604_2605delAG, and two substitutions, NM_001135243.2:c.2575C>T and NM_001135243.2:c.4111G>T. All three reported mutations are in hotspot regions (exons 16 and 24) and result in a shortened protein. These pathogenic variants are not observed in relatives of the examined probands, which indicates that all are de novo mutations. All genetic alterations identified within our work are presented in Table 2.

**Table 2:** Mutations identified in three unrelated TCS patients.

Patient	Exon	Exon variant	Predicted effect	Status	Classification	Geographic origin	Reference
1	16	NM_001135243.2 (TCOF1_v001): c.2604_2605delAG	NM_001135243.2 (TCOF1_i001): p.Gly869Glufs*3	Hetero	Pathogenic	Caucasian	de novo
2	16	NM_001135243.2 (TCOF1_v001): c.2575C>T	NM_001135243.2 (TCOF1_i001): p.Gln859*	Hetero	Pathogenic	Caucasian	de novo
3	24	NM_001135243.2 (TCOF1_v001): c.4111G>T	NM_001135243.2 (TCOF1_i001): p.Glu1371*	Hetero	Pathogenic	Caucasian	de novo

The first descriptions of TCS date back to the early twentieth century; however, a full understanding of its genetic origins requires further study. Research has confirmed that in addition to *TCOF1*, mutations in *POLR1C*, *POLR1D*, and *POLR1B*^4,5^ can lead to TCS. However, for 8–11% of patients, the pathogenesis of the disorder remains uncertain^18^. This may indicate the involvement of other genes or other mechanisms leading to TCS. Duan et al.^20^ presented the hypothesis that oxidative stress may be one of the causes of TCS.

Molecular diagnosis is important in rare disorders and is used in prenatal and postnatal screening. Other disorders, such as Oculoauriculovertebral spectrum, Miller syndrome and Nager syndrome, possess phenotypic features similar to those of TCS, making the diagnosis of TCS difficult. The most effective tool to further explore mutations and genes associated with the origin of TCS would be whole-exome sequencing^18^.

## HGV database

The relevant data from this Data Report are hosted at the Human Genome Variation Database at 10.6084/m9.figshare.hgv.3086, 10.6084/m9.figshare.hgv.3089, and 10.6084/m9.figshare.hgv.3092.
